# Opt-Out Panel Testing for HIV, Hepatitis B and Hepatitis C in an Urban Emergency Department: A Pilot Study

**DOI:** 10.1371/journal.pone.0150546

**Published:** 2016-03-11

**Authors:** Sarah O’Connell, Darren Lillis, Aoife Cotter, Siobhan O’Dea, Helen Tuite, Catherine Fleming, Brendan Crowley, Ian Fitzgerald, Linda Dalby, Helen Barry, Darragh Shields, Suzanne Norris, Patrick K. Plunkett, Colm Bergin

**Affiliations:** 1 Department of Genito-Urinary Medicine and Infectious Disease, St James’s Hospital, Dublin, Ireland; 2 Emergency Medicine Department, St James’s Hospital, Dublin, Ireland; 3 Hepatology Department, St James’s Hospital, Dublin, Ireland; 4 Infectious Diseases Department, Galway University Hospital, Dublin, Ireland; 5 Microbiology Department, St James’s Hospital, Dublin, Ireland; 6 Department of Clinical Medicine, Trinity College Dublin, Dublin, Ireland; Centers for Disease Control and Prevention, UNITED STATES

## Abstract

**Objectives:**

Studies suggest 2 per 1000 people in Dublin are living with HIV, the level above which universal screening is advised. We aimed to assess the feasibility and acceptability of a universal opt-out HIV, Hepatitis B and Hepatitis C testing programme for Emergency Department patients and to describe the incidence and prevalence of blood-borne viruses in this population.

**Methods:**

An opt-out ED blood borne virus screening programme was piloted from March 2014 to January 2015. Patients undergoing blood sampling during routine clinical care were offered HIV 1&2 antibody/antigen assay, HBV surface antigen and HCV antibody tests. Linkage to care where necessary was co-ordinated by the study team. New diagnosis and prevalence rates were defined as the new cases per 1000 tested and number of positive tests per 1000 tested respectively.

**Results:**

Over 45 weeks of testing, of 10,000 patient visits, 8,839 individual patient samples were available for analysis following removal of duplicates. A sustained target uptake of >50% was obtained after week 3. 97(1.09%), 44(0.49%) and 447(5.05%) HIV, Hepatitis B and Hepatitis C tests were positive respectively. Of these, 7(0.08%), 20(0.22%) and 58(0.66%) were new diagnoses of HIV, Hepatitis B and Hepatitis C respectively. The new diagnosis rate for HIV, Hepatitis B and Hepatitis C was 0.8, 2.26 and 6.5 per 1000 and study prevalence for HIV, Hepatitis B and Hepatitis C was 11.0, 5.0 and 50.5 per 1000 respectively.

**Conclusions:**

Opt-out blood borne viral screening was feasible and acceptable in an inner-city ED. Blood borne viral infections were prevalent in this population and newly diagnosed cases were diagnosed and linked to care. These results suggest widespread blood borne viral testing in differing clinical locations with differing population demographic risks may be warranted.

## Introduction

Screening for infectious diseases including HIV, Hepatitis B (HBV) and Hepatitis C (HCV) offers a health benefit to the individual and prevents onward transmission. These viruses are world-wide public health problems resulting in a significant impact on healthcare resource utilisation and costs. [1,2] Furthermore these infections disproportionately affect socially marginalised groups.

In 2014, 377 people were newly diagnosed with HIV-1 infection in Ireland, representing an 11% increase from 2013. [3] A HIV prevalence rate of over 2 per 1000 among 15–59 year olds in the Dublin area has previously been reported. [4] In 2013 there were 429 new diagnoses of HBV reported in Ireland, where the HBV prevalence rate is thought likely to be <0.5%. [5, 6] 786 cases of HCV were reported in 2013 in Ireland and the HCV prevalence rate is estimated to be between 0.5 and 1.2%. [7] According to ECDC data, despite falling rates in recent years, Ireland has one of the highest rates of new hepatitis B and C notifications in Europe. [8] Furthermore, it is estimated that the majority of patients infected with HCV in Ireland remain undiagnosed. [9]

There continues to be significant morbidity and mortality related to late diagnosis of HIV. In Ireland, 49.5% of new HIV cases in 2013 presented late, with 28% having a CD4 cell count of less than 200/mm3. [10] Associated with late diagnoses are significant costs; the direct medical costs for HIV care in the first year after diagnosis are twice as high as those with a CD4 count < 350. [11] The benefits of an earlier diagnosis of HIV include earlier access to treatment, reduced mortality, reduced risk of cardiovascular diseases and malignancies and overall improved outcomes. Furthermore, in 2006 it was estimated that 50% of new HIV infections in the US were transmitted by the 25% of HIV-positive individuals unaware of their status and more recently the CDC estimate 12.8% of those in the US with HIV infection are unaware of their HIV status. [12, 13]

Complications of chronic HBV and HCV infection include cirrhosis, decompensated liver disease, hepatocellular carcinoma and death. In an era of newly developed successful HCV therapies and the introduction of the Irish Health Service Executive (HSE) Service Plan for 2015 that provides a financial commitment to treat those with HCV infection, the requirement to test and diagnose those with unknown HCV infection has vastly increased in recent times. The role of anti-viral therapies to suppress HBV infection has also clearly been demonstrated. [14] Early treatment of HBV and HCV infection can prevent disease progression, the complications of which represent a significant burden of care to the Irish healthcare system.

CDC guidelines in the USA recommend an opt-out HIV screening approach. In the UK it is recommended that HIV testing is considered where HIV prevalence rates exceed 2/1000. [15] In the US, the CDC recently recommended one-time HCV screening of all persons born between 1945 and 1965, in addition to risk factor based screening already in place. [16] CDC HBV testing guidelines suggest testing those with exposure risk factors to HBV. [17]

In Ireland risk-based testing for HIV, HBV and HCV is most commonly offered. Despite the known prevalence in Dublin, universal HIV and HBV screening is performed on an opt-out basis only in antenatal care, sexual health clinics and for blood donors. Common risk demography exists for HCV acquisition in migrants, men who have sex with men and persons who inject drugs (PWID). Significant rates of HCV have been reported amongst PWID in the Dublin maternity hospitals also. [18,19] Despite these factors, only risk based targeted HCV screening strategies are operational in Ireland and in most maternity hospitals. The universal screening of migrants has not yet been undertaken and overall there has been no integrated programme to screen patients for multiple blood-borne viral (BBV) infections with panel testing in a healthcare setting.

The offer of HIV testing in healthcare settings that do not routinely test for BBV infections including Out-Patient Clinics, General Practice, Acute Care Units and Emergency Departments has been shown to be acceptable and feasible. [20] Similar screening strategies include a universal point of care testing approach in an acute admission unit; this appeared to be an effective, feasible, acceptable and low cost approach to HIV screening. [21] The emergency department (ED) is a desirable target for HIV testing within hospitals as it serves a high-throughput population of diverse attendees. Our ED serves a population with a very high diagnosed prevalence of HIV (2.25 per 1000) and this busy ED has 46,000 attendances per year.

## Methods

This was a cross-sectional study conducted at a large urban Emergency Department in Dublin, Ireland. Collaboration between the Departments of Genito-Urinary and Infectious Diseases, Emergency Medicine, Hepatology and Microbiology allowed for the opt-out screening of all suitable patients who had bloods taken as part of routine clinical care. Nursing and Medical staff in the ED attended didactic teaching sessions and workshops provided by the Infectious Disease and Emergency Department study team to provide background information for the pilot study and to explain the testing protocol.

All patients over the age of 18 with the capacity to consent were included, as outlined in ethical approval granted by our local ethics committee. Posters were placed in the waiting room and patient information leaflets were made available at various care stages throughout the Emergency Department, including at reception and at triage. Patient information leaflets were translated into the 7 most common languages. A contact number for the responsible study team member was provided on these and on separate slips for those patients who had further questions for the study team or to find out their results.

**Fig 1:**
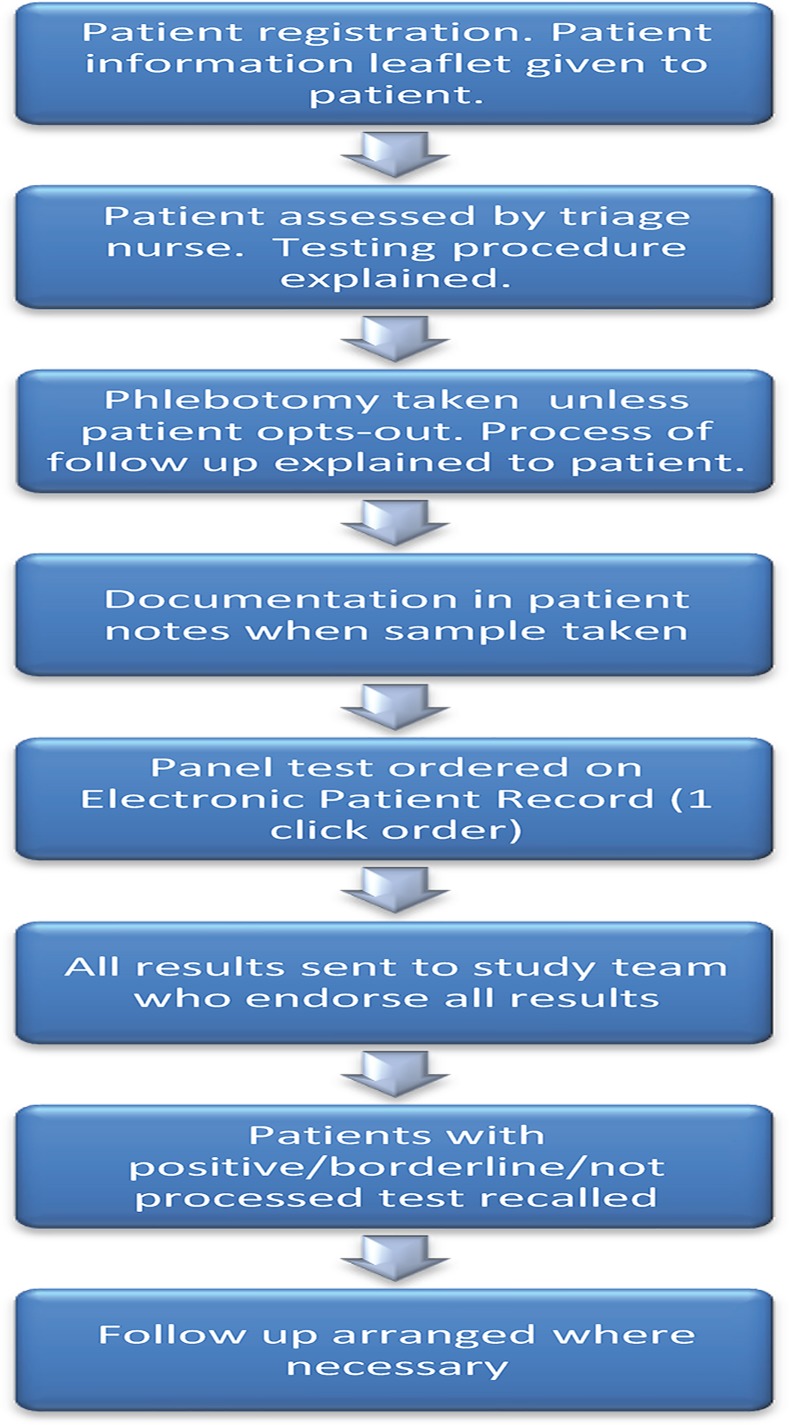
Patient Testing and Results Process.

Patients were advised that an extra serum sample would be taken when undergoing phlebotomy as part of routine clinical care at no extra cost, and this sample would be tested for HIV, Hepatitis B and Hepatitis C viral infection. The patient was given the option to opt-out following a set time period of 20 minutes to consider his/her options. (Fig 1) Verbal consent was obtained in all cases in line with good clinical practice and current international clinical guidelines [22]. Written consent was deemed unnecessary as stated in these guidelines and is no longer part of routine clinical practice. When a study blood sample was taken this was recorded in the Emergency Department patient case notes.

Patients were informed that they would be contacted 3 working days after their initial ED visit, if the blood test taken was reactive. Patients were also informed that they would not be contacted if results were negative.

Appropriate ethical approval was obtained from the St James’s Hospital/Adelaide and Meath, National Children’s Hospital (SJH/AMNCH) Research Ethics Committee. (REC reference 2014/01) The consent process was approved by this committee.

Given potential common risk demography for all three infections, panel testing for all three infections was performed in the possible scenario that a patient disclosed one blood borne viral infection to the healthcare staff member at the time of screening, or where the patient had study bloods taken at an earlier point in the study.

Weekly progress updates were sent by the study team the ED staff to inform them of study blood uptake and details of positive tests found. Furthermore, information and Q and A sessions were provided for the ED staff throughout the project.

Results governance and delivery was managed by the study team and patients requiring follow up bloods or with a reactive blood test were contacted using contact details provided at ED reception. Follow up bloods were taken at the study facility and appropriate patients were linked to care. Those who had previously disengaged from care were also identified in the study and linkage back to care was co-ordinated by the study team. Patient demographics for those who had a positive test were subsequently captured on electronic and paper chart review.

Taking into account a range of previous opt-in rates (23% - 66%) reported for HIV testing in Emergency Departments [23,24, 25], and a previous study which showed a higher patient offer rate with an opt-out approach,[26] target uptake rate was set at greater than 50%. Uptake rate was defined as the number of study bloods taken when the patient was undergoing phlebotomy as part of routine clinical care, proportional to the total number of bloods taken in the Emergency Department over the study period.

New diagnosis rate and study prevalence were defined as the number of new cases per 1000 tested and number of positive tests per 1000 tested for BBV respectively.

The primary aim of this study was to assess the feasibility and acceptability of this approach of panel testing for HIV, HBV and HCV in a busy urban ED where opt-out testing was performed on patients having bloods done as part of routine clinical care.

The secondary aim of this study was to determine the sero-prevalence of these three viral infections in this ED and to determine the new diagnosis rate of new viral infections. New and previously known and disengaged patients were linked back to care for early treatment to prevent morbidity/mortality and to prevent onward transmission.

## Results

10,000 serum samples were tested for HIV antibody/antigen, hepatitis B surface antigen and hepatitis C antibody from March 2014 to January 2015. A sustained cumulative target testing uptake rate of 50.1% was obtained. Of all ED blood samples taken, the proportion of study bloods taken was greater than 80% on specific days selected, where uptake of study bloods was examined using Emergency Department patient notes.

**Fig 2:**
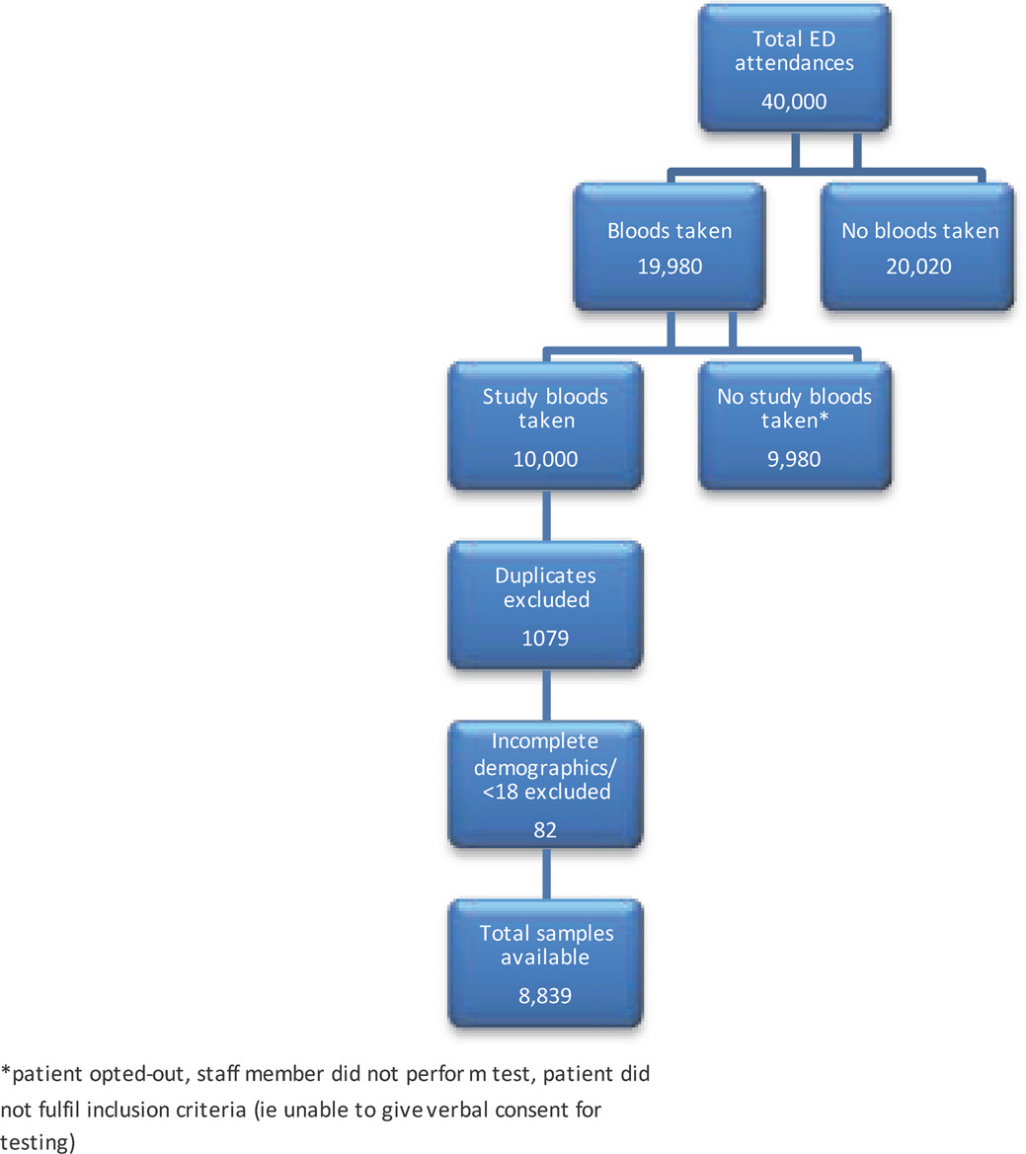
Description of Patient Numbers Tested.

1079 subjects had a sample tested greater than once during the study period. 74 subjects under the age of 18 and 8 subjects with incomplete demographics were excluded from analysis. Following exclusion of these patients and removal of duplicates, a total of 8,839 individual patient test results were available for analysis. (Fig 2) Median age (IQR) for this group tested was 45 (32,66). Age range was 18–102 years. 4463 (50.4%) were male subjects.

### HIV

97 subjects who underwent testing had a positive HIV test. Age range (years) was 20–60. Median (IQR) years was 39(33,43). 68 patients (61.8%) were male. 7 of these patients were new diagnoses and 90 of these patients were previously known. 89 (98.8%) of patients with previously known infection were linked to care at the time of testing, and one further patient was re-linked to care as result of study team intervention.

**Fig 3:**
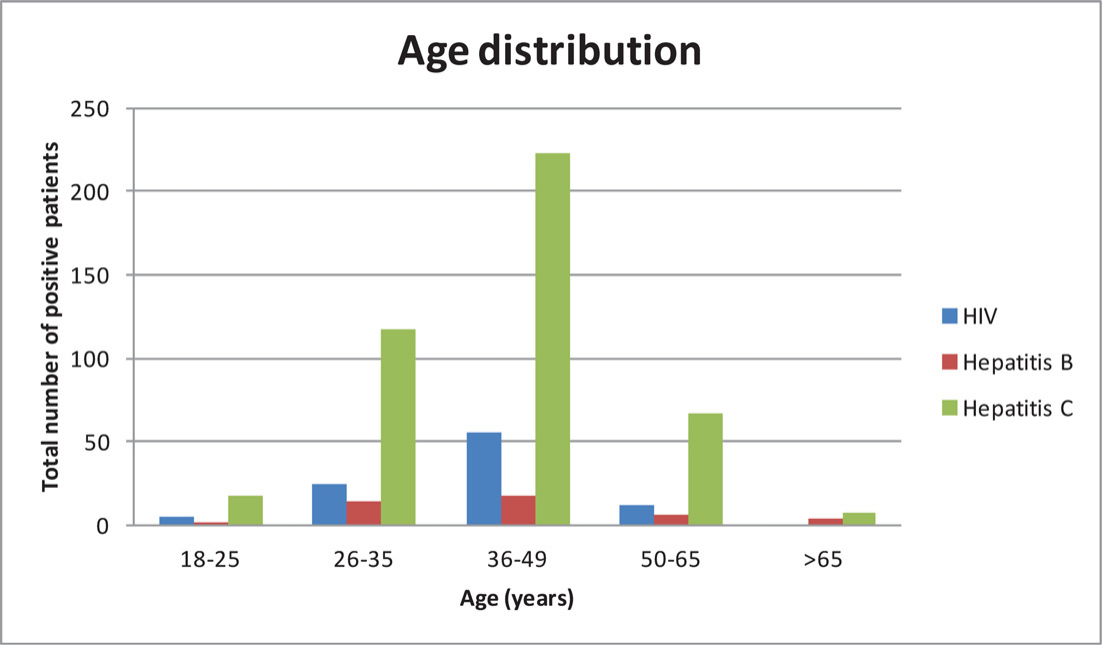
Positive Patient Age Distribution.

Of the 7 newly diagnosed patients, 4 were male, age range was 23–51 and median (IQR) age was 29.5(38,43.5). (Fig 3) Mode of acquisition included 3 MSM, 3 Irish female patients with heterosexual contact with a person from a country of high prevalence and 1 male with a history of previous intra-venous drug use. 4 (57.1%) of these patients presented to the ED with no clinical indicators for HIV infection. 3 (42.9%) presented with clinical indicators including *Pneumocystis Jirovecii* pneumonia (n = 2) and pyrexia of unknown origin (n = 1), with a subsequent diagnosis of Multicentric Castleman’s disease. Five of these patients presented at a late point in their illness, with a Cd4 count of less than 350 cells/mm3. Two of these patients had a CD4 count of less than 50 cells/mm3 at presentation, one of less than 200 cells/mm3 and the remaining two had a CD4 count of less than 350 cells/mm3. One patient was experiencing HIV seroconversion at the time of testing based on viral load result, avidity and HIV antibody results. HIV -1 viral load measured for this patient was greater than 10 million copies/mL, posing a very high risk of onward transmission. 1 patient (MSM) had previously tested for HIV; this was negative 1 year prior to diagnosis. 5 patients have been successfully commenced on ART; a further 2 patients have plans to start in the near future, in the setting of interim results of a large-scale randomised clinical trial that recently showed significant clinical benefits for those patients commenced on ART at an earlier point in their illness, thus supporting the US recommendation that all asymptomatic HIV positive patients take ART irrespective of CD4 count. [27] (Table 1)

**Table 1:** Demographics of Positive Patients.

	HIV	Hepatitis B	Hepatitis C
	N (%)	N (%)	N (%)
**Age**			
**New**	**7**	**20**	**58**
*Range*	23–51	29–78	18–69
*Median (IQR)*	29.5 (38,43.5)	44 (34,57)	25 (36,49)
**Known**	**90**	**23**	**373**
*Range*	20–60	21–72	18–81
*Median (IQR)*	47.5 (33,44.5)	42 (33.5,45)	39 (34,46)
**Gender**			
**New**	**7**	**20**	**58**
*Male*	4 (57.1)	16 (80)	47 (81)
*Female*	3 (42.9)	4 (20)	11 (19)
**Known**	**90**	**23**	**373**
*Male*	57 (63.3)	16 (69.6)	235 (63)
*Female*	33 (36.7)	7 (30.4)	138 (37)
**Mode of Acquisition**			
**New**	**7**	**20**	**58**
*MSM*^*a*^	3 (42.8)	1 (5)	0
*IVDU*^*b*^	1 (14.3)	1 (5)	37 (63.8)
*HS*^*c*^	3 (42.8)	0	2 (3.4)
*NRI*^*d*^	0	9 (45)	14 (24.1)
*Vertical/COHP*^*e*^	0	9 (45)	3 (5.1)
*Nasal DU*^*f*^	0	0	2 (3.4)
**Known**	**90**	**23**	**373**
*IVDU*^*b*^	48 (53.3)	5 (21.8)	333 (89.3)
*HS*^*c*^	12 (13.3)	13 (56.5)	5 (1.3)
*Blood Transfusion*	0	1 (4.3)	4 (1)
*Anti D*	0	0	5 (1.3)
*NRI*^*d*^	0	2 (8.7)	21 (5.6)
*Nasal DU*^*f*^	0	0	1 (0.3)
*Vertical*^*e*^	0	1 (4.3)	2 (0.5)
*Haemophilia*	0	0	1 (0.3)
*MSM*^*a*^	30 (33.3)	1 (4.3)	1 (0.3)
**Active IVDU**			
**New**	**7**	**20**	**58**
*Active IVDU*^*b*^	1 (14.3)	1 (5)	37 (63.8)
**Known**	**90**	**23**	**373**
*Active IVDU*^*b*^	17 (18.8)	0 (0)	135 (40.5)
**High Risk Postal Codes**			
**New**	**7**	**20**	**58**
*Dublin area*	7 (100)	18 (90)	54 (93.1)
**Known**	**90**	**23**	**373**
*Dublin area*	63 (70)	19 (82.6)	221 (59.2)
**Homelessness**			
**New**	**7**	**20**	**58**
*No fixed abode*	2 (28.5)	1 (5)	19 (32.7)
**Known**	**90**	**23**	**373**
*No fixed abode*	20 (22.2)	3 (13)	125 (33.5)

^a^MSM: Men who have sex with men

^b^IVDU: Intravenous drug users

^c^HS: Heterosexual

^d^NRI: No risk identified

^e^COHP: Country of high prevalence

^f^Nasal DU: Nasal drug use

Of those with previously diagnosed HIV infection, 70 (77.6%) were Irish. Other countries of origin included United Kingdom (n = 1), Brazil (n = 5), Greece (n = 1), Latvia (n = 1), Lithuania (n = 1), Russia (n = 1), Sub-saharan Africa (n = 7), Poland (n = 2) and Pakistan (n = 1).

49 (50.5%) and 2 (2%) patients were co-infected with hepatitis C and B viral infection respectively. All these patients were aware of their co-infection status.

Emergency Department new diagnosis and prevalence rates for HIV infection were 0.8 and 11 per 1000 respectively. Patients were recalled and linked to care where appropriate. (Table 2)

**Table 2:** Description of Linkage to Care.

N = 8,839	N (%)
**Total HIV+ cases**	**97**
**New HIV+ cases**	**7**
*Now linked*	7 (100)
*Unlinked*	0 (0)
**Known HIV+ cases**	**90**
*Linked*	90 (100)
*Unlinked*	0 (0)
**Total hepatitis B sAg+ cases**	**44**
**New hepatitis B sAg+ cases**	**20**
*Now linked*	19 (95.0)
*Linkage ongoing*	1 (5.0)
**Known hepatitis B sAg+ cases**	**23**
*Linked*	23 (100)
**Undetermined new/known hepatitis B sAg+ve**	**1 (2.27)**
**Total hepatitis C Ab+ cases**	**447**
**New hepatitis C Ab+ cases**	**58**
*Now linked*	43 (74.1)
*Linkage not required (PCR–ve)*	11 (18.9)
*Unlinked*	4 (6.89)
**Known hepatitis C Ab+ cases**	**373**
*Linked*	202 (54.1)
*Relinked*	80 (21.4)
*Relinkage not required (PCR–ve)*	64 (17.1)
*Unlinked*	27 (7.2)
**Undetermined new/known hepatitis C Ab+ve**	**16 (3.5)**

### Hepatitis B

A total of 44 patients had a positive blood test for hepatitis B surface antigen. 23 of these patients were known and 20 were new diagnoses. One further patient is currently lost to follow up. Of the 20 newly diagnosed patients, with chronic hepatitis B infection, 16 were male, age range was 29–78 and median (IQR) age was 44(34–57). (Fig 3) 6(30%) of these patients are from Ireland, other countries of origin included Afghanistan (n = 1), China (n = 3), Romania (n = 3) and Brazil (n = 1), Eastern Europe (n = 2), Pakistan (n = 1), Phillipines (n = 1) and sub-saharan Africa (n = 2). Mode of acquisition included vertical transmission (n = 9), MSM (n = 1), IVDU (n = 1) and a further 9 patients (45%) had no identifiable risk when a full clinical history was taken at subsequent out-patient assessment. (Table 1)

8 (34.8%) of those with previously diagnosed HBV infection were Irish. Other countries of origin included Italy (n = 1), Brazil (n = 1), Latvia (n = 1), Sub-Saharan Africa (n = 8), Thailand (n = 1) and Pakistan (n = 3).

ED new diagnosis and prevalence rates for chronic hepatitis B infection were 2.26 and 5 per 1000 respectively. Patients were recalled and linked to care where appropriate. (Table 2)

### Hepatitis C

447 patients had a positive blood test for hepatitis C antibody. 58 of these tests were newly diagnosed infection and 373 were previously diagnosed. A further 16 are currently lost to follow up, have not received their results and attempts to actively contact these patients are being pursued.

Of the newly diagnosed group (n = 58), 15% (n = 12) had a negative PCR during study follow up. Age range for this group was 18–69, median (IQR) age was 25(36,49). (Fig 3) 47(81%) were male. Country of origin included 52 Irish, Morocco (n = 1), Pakistan (n = 1), China (n = 1), Asian (n = 1) and Latvian (n = 2).

Of the 43 patients with newly diagnosed hepatitis C linked to care, 32 had a history of intravenous drug use, 1 with a history of nasal cocaine use, 6 had no risk identified on assessment, 1 reported due to blood transfusion, 1 nosocomial acquisition, 2 heterosexual risk and 39 (93%) were Irish. Of those 32 patients who had a history of intravenous drug use, 12 were actively injecting drugs, 18 previously injected drugs and 2 had unknown active injection drug use status. 16 (50%) of these patients with a history of intravenous drug use were receiving methadone replacement therapy. (Table 1)

364 (97.6%) of those with previously diagnosed HCV infection were Irish. Other countries of origin included United Kindgom (n = 1), Afghanistan (n = 1), Latvia (n = 1), Poland (n = 2), Ukraine (n = 1), Congo (n = 1), China (n = 1) and country of origin for one patient is unknown.

3 newly diagnosed patients seroconverted to hepatitis C viral infection during the study duration, with an initial negative hepatitis C antibody test followed by newly diagnosed positive during the study period on re-attendance to the Emergency Department. 2 of these patients carried risk of intravenous drug use for acquisition and 1 patient has no identifiable risk for acquisition. 1 newly diagnosed patient was treated for HCV 10 years previously and after a subsequent positive PCR was diagnosed with re-infection as a result of ongoing intravenous drug use.

ED new diagnosis and prevalence rates for hepatitis C viral infection were 6.5 and 50.5 per 1000 respectively. Patients were recalled and linked to care where appropriate. (Table 2)

## Discussion

This study demonstrates the novel use of panel testing as a method of screening for blood-borne viruses including HIV, hepatitis B and hepatitis C viral infection in a busy urban ED. No previous studies have been conducted examining this method as a screening strategy.

A high uptake rate was seen from an early point in the study, with 50.1% of all bloods in the ED taken over the study period containing an extra sample for viral screening. ED staff members reported it was easier to take the bloods on a regular basis instead of consideration of risk before the patient underwent phlebotomy. The medical teams at our hospital gave feedback that with the process of blood-borne viral screening being performed at the stage of presentation to the hospital and this allowed for a faster assessment process and formulation of differential diagnosis by including immunosuppression or hepatitis as a factor in clinical decision making.

A broad range of ages were tested and as testing was undertaken on an opt-out basis, patient risk was not considered prior to screening, thus the assumption is held that all risk groups were possibly screened.

A high Emergency Department study prevalence was seen for all three viral infections, with a positive HIV blood test in 1.1% of attendees having study bloods taken. This prevalence rate is much higher than the results of previous work that determined the rate of those living with HIV in the greater Dublin area to be at least 0.2% and suggests that HIV screening should be performed as routine in those undergoing venepuncture or accessing secondary care. Furthermore, 5% of all attendees undergoing study bloods had a positive hepatitis C antibody test and a significant number of HIV/Hepatitis C co-infection was noted. Both these rates reflect our inner-city attending cohort and high prevalence of intravenous drug use in this group.

On the basis of these results, to advocate patient safety and to reduce the risk of onward transmission, we have implemented this practice as standard of care, in partnership with the HSE Acute Hospitals Division, National Social Inclusion Unit and the Population Health and Wellbeing Directorate.

This study had some limitations. Due to the study design of opt-out testing as part of routine clinical care in a busy ED and limitations with our electronic medical record system, we were unable to ascertain a refusal rate of HIV tests offered, or to obtain patient feedback to establish acceptability of patients at the time of routine screening. Furthermore, risk demography of patients was not routinely collected at the time of testing, thus electronic patient records were relied upon to collect this risk demography subsequent to screening. ED prevalence and new diagnosis rates reflect those who had study bloods done only, thus these reported figures may be an over–representation of viral infection prevalence in the ED attendees. Conversely, the HIV new diagnosis rate found was lower than those found in previous studies [28,29]. This study design incorporated an opt-out approach, where patients having phlebotomy done as part of clinical care were given the option to opt out of BBV testing. In previous studies, an “opt-in” approach has been utilised, where patients are approached and offered the tests, and different testing methods have been used including salivary point of care tests in these studies. It is possible that due to the exclusion of greater than 50% of ED attendees over the study period who did not have bloods taken, the HIV new diagnosis rate found was lower than if this cohort had undergone testing.

A very small proportion of those patients testing positive for blood borne viral infection were aged >65. Given the high cost of such a screening approach, it is possible that costs may have been decreased by excluding this older population. A detailed cost analysis will be performed to assess the cost efficiency of performing panel testing for HIV, hepatitis B and C viral infections in this setting will address this question.

As part of the extension of the proposed NaTIve (National Viral Testing Initiative) project, further studies examining the sero-prevalence rates in both targeted and non-targeted settings in areas of differing demographics will be performed. Following this, a detailed cost-efficiency study will be performed to examine these testing approaches.

## Conclusion

Within a large urban Emergency Medicine Department in a tertiary referral centre, it was possible to achieve an uptake rate of 50% in an HIV, hepatitis B and C opt-out testing pilot study. Over a 10 month period, 7 new HIV diagnoses were made, demonstrating the importance of testing in the ED to reduce onward transmission. Patients diagnosed had mainly late HIV diagnoses and were not all from high risk-groups, suggesting scope for further universal testing. This study enabled linkage to care of new individuals for early treatment in an era of early treatment for HIV and newly developed successful therapies for HCV with high success rates.

The impact of this study has positive public health implications both at an individual patient and at population health level. It has raised awareness about HIV, HBV and HCV testing and its clinical indicators. Given the opt-out nature of the test, stigma has potentially been removed from the process of testing, for both the patient and also the staff member. Furthermore, those patients unaware of their diagnosis are at a significant health advantage by being diagnosed at an earlier stage in their illness. Once diagnosed and treated appropriately, rates of transmission will decrease. The strategy of linking treatment to testing (Test and Treat) has proved an effective public health intervention for HIV infection and may become part of the ambitious Department of Health public health intervention to eradicate HCV. While current European guidelines recommend routine commencement of ART for HIV at a CD4 count of greater than 350 cells/mm^3^, interim results of a large-scale randomised clinical trial recently showed significant clinical benefits for those patients commenced on ART at an earlier point in their illness, thus supporting the US recommendation that all asymptomatic HIV positive patients take ART irrespective of CD4 count and the strategy of linking treatment to testing. [30]

While previous studies have involved opportunistic HIV testing alone in non-traditional environments including Emergency Medicine Departments, no study to date has looked at the benefits of opt-out panel testing- where all 3 viruses are tested from a single serum sample. Particularly of note, of those who tested positive for HIV infection, our pilot study showed that almost half those patients with HIV infection had co-infection with Hepatitis C, thus to screen for one virus alone runs the risk of missing other infections at the time of screening.

This pilot study has not only offered patients a unique opportunity to be tested but also to be linked back to care. This study has provided us with valuable local population prevalence data that will inform blood-borne virus testing practice in the near future.
